# Elevated Plasma Soluble Sema4D/CD100 Levels Are Associated with Disease Severity in Patients of Hemorrhagic Fever with Renal Syndrome

**DOI:** 10.1371/journal.pone.0073958

**Published:** 2013-09-10

**Authors:** Bei Liu, Ying Ma, Jing Yi, Zhuwei Xu, Yu Si Zhang, Chunmei Zhang, Ran Zhuang, Haitao Yu, Jiuping Wang, Angang Yang, Yun Zhang, Boquan Jin

**Affiliations:** 1 Department of Immunology, The Fourth Military Medical University, Xi’an, China; 2 Department of Blood Transfusion, Xijing Hospital, The Fourth Military Medical University, Xi’an, China; 3 Department of Infectious Disease, Tangdu Hospital, The Fourth Military Medical University, Xi’an, China; University of Kansas Medical Center, United States of America

## Abstract

**Background:**

Hantaan virus (HTNV) could cause a severe lethal hemorrhagic fever with renal syndrome (HFRS) in humans. Despite a limited understanding of the pathogenesis of HFRS, the importance of host-related immune responses in the pathogenesis of HFRS has been widely recognized. CD100/Sema4D has been demonstrated to play an important role in physiological and pathological immune responses, but the functional role of CD100 in infectious diseases has only been inadequately reported. The aim of this study was to investigate the pathological significance of CD100 in patients after HTNV infection.

**Methodology/Principal Findings:**

Blood samples were collected from 99 hospitalized patients in Tangdu Hospital and 27 health controls. The level of soluble CD100 (sCD100) in plasma were quantified by ELISA and the relationship between sCD100 and the disease course or severity were analyzed. The expressions of membrane CD100 on various subpopulations of peripheral blood mononuclear cell (PBMC) were analyzed by flow cytometry. The results showed that sCD100 level in acute phase of HFRS was significantly higher in patients than that in healthy controls (*P*<0.0001) and the sCD100 level declined in convalescent phase. Multivariate model analysis showed that platelet count, white blood cell count, serum creatinine level and blood urea nitrogen level were associated with sCD100 levels and contributed independently to the elevated sCD100 levels. The expression of membrane CD100 on PBMCs decreased in the acute phase of HFRS patients compared with that of the normal controls and recovered in the convalescent phase.

**Conclusions:**

We reported the elevated level of plasma sCD100 in HFRS patients and the elevated level might be a result from the shedding of membrane CD100 on PBMC. The elevated level of sCD100 was associated with disease severity, suggesting that sCD100 might be a cause or a consequence of progression of HFRS. The underlying mechanisms should be explored further.

## Introduction

Hantaan virus (HTNV), which belongs to the genus *Hantavirus* of the family *Bunyaviridae*, could cause a severe lethal hemorrhagic fever with renal syndrome (HFRS) in human. More than 100,000 cases of HFRS, over 50% of which were documented in mainland of China, occurr annually worldwide with a mortality rate of 2-10% [1,2]. People with HFRS are clinically characterized by sudden fever, hemorrhage, thrombocytopenia, and acute renal failure. Typically, the course of HFRS undergoes five sequential stages: febrile, hypotensive, oliguric, diuretic, and convalescent. Although the importance of immune responses after HTNV infection, including immune complexes, complement activation, B cell response, T cell response and HTNV-induced cytokine production, has been widely recognized [2–6], the pathogenesis of HFRS is far from being completely understood.

The 150 kDa transmembrane protein CD100/Sema4D belongs to group IV of the semaphorin family, the first known semaphorin identified in the immune system [7], and is involved in several aspects of both humoral and cellular immunity [8–13]. CD100 exists in both membrane-bound and soluble forms. The membrane CD100 is preferentially expressed on T cells and weakly on B cells and on antigen presenting cells (APC) [8,14]. Cellular activation can cause the release of sCD100 and sCD100 is demonstrated to retain biological activities such as acting as a costimulator for CD40-induced B-cell proliferation and Ig production and affecting pro-inflammatory cytokines production by APCs [10,13]. There are two types of receptors that CD100 used to bind: Plexin-B1 mainly expressed in nonlymphoid tissues [15] and CD72 mainly expressed in the immune system [8].

Accumulating evidence indicates that CD100 plays an important role in physiological and pathological immune responses. CD100^-/-^ mice are viable, but show defective T cell priming and B cell responses, whereas adaptive immune responses are significantly enhanced in CD100 transgenic mice [11,14]. CD100 is also believed to be involved in some clinical diseases. Soluble CD100 was detected in the spinal cords of patients with central nervous system inflammatory disease [16] and in sera of patients with autoimmune disease [17], suggesting the potential role of sCD100 in the development and/or maintenance of these diseases. Recently, Eriksson et al investigated the consequence of HIV-1 infection on CD100 expression of T cells and they observed a subset of CD8^+^ T cell lacking of membrane CD100 with decreased functional capacity. Their findings suggested that loss of CD100 expression would probably lead to dysfunctional immunity in HIV-1 infection [18]. However, knowledge of the functional role of CD100 in infectious disease is still limited. Whether this pathogenetic role of CD100 could extend to other acute infectious diseases mediated by immune responses is also unclear.

In terms of the important role of CD100 in immune response, we hypothesized that CD100 may also involved in the pathogenesis of HFRS. We focused on two questions: 1) whether the changes of CD100 expression and sCD100 release after HTNV infection exist, and 2) whether these changes would correlate with the development and severity of the disease. Plasma and peripheral blood mononuclear cell (PBMC) samples from 99 HFRS patients and 27 health controls were collected. The plasma sCD100 levels and membrane CD100 expressed on PBMCs from HFRS patients of different severities and in different disease stages were quantified. The relationships between sCD100 and the disease course as well as disease severity-indicating parameters were also analyzed.

## Methods

### Ethics Statement

The study was approved by the Institutional Review Board of the Fourth Military Medical University. Written informed consent was obtained directly from each adult subject. Parents and guardians of participating children had the aims of the research explained, and written consent was obtained from children participants’ guardians on the behalf of the children for collection of samples and subsequent analysis.

### Patients

Enrolled in the study were 99 hospitalized HFRS patients in Tangdu Hospital of the Fourth Military Medical University (Xi’an, China) from October 2009 to December 2011 (see Table 1). The clinical diagnosis of HFRS was confirmed serologically by the detection of IgM and IgG antibodies to HTNV nucleocapsid protein. Twenty-seven healthy volunteers were also included in the study as normal control. Plasma samples were collected from the patients during hospitalization. All samples were frozen at −20°C until use. The HFRS disease severity (including mild, moderate, severe and critical) was classified on the basis of clinical and laboratory parameters used in the diagnostic criteria for HFRS in China as (1) mild, mild renal failure without an obvious oliguric stage; (2) moderate, obvious symptoms of uremia, effusion (bulbar conjunctiva), hemorrhage (skin and mucous membrane) and renal failure with a typical oliguric stage; (3) severe, severe uremia, effusion (bulbar conjunctiva and either peritoneum or pleura), hemorrhage (skin and mucous membrane), and renal failure with oliguria (urine output, 50–500 mL/day) for ≤5 days or anuria (urine output,<50 mL/day) for ≤ 2 days; and (4) critical, cases with ≥1 of the following symptoms: refractory shock, visceral hemorrhage, heart failure, pulmonary edema, brain edema, severe secondary infection and severe renal failure with either oliguria (urine output, 50–500 mL/day) for > 5 days or anuria (urine output, <50 mL/day) for >2 days, or a blood urea nitrogen level of > 42.84 mmol/L [19]

**Table 1:** Clinical Characteristics of HFRS Patients.

	Mild	Moderate	Severe	Critical
Demographic characteristics				
Patient number	17	25	29	28
Sample number	26	49	64	56
age (years)	28 (15-35)	34 (24-46)	47 (35-54)	45 (33-60)
Males (%)	88.2	76.0	72.4	85.7
Clinical parameters at febrile or hypotensive stage				
sCD100 (ng/mL)	29.5 (17.6-51.4)	35.2 (16.3-46.7)	49.3 (25.0-52.3)	59.2 (52.2-71.4)
White Blood Cell count（×10^3^/µL)	10.8 (7.8-16.9)	11.3 (5.7-22.1)	12.2 (4.7-23.7)	21.0 (15.0-33.0)
Platelet Count（×10^3^/µL)	59.5 (50.3-117)	69.5 (37.8-127.5)	35.5 (19.5-62.3)	17.9 (13.1-41.0)
blood urea nitrogen（µmol/L)	8.9 (5.0-14.0)	9.3 (6.1-16.6)	13.4 (5.8-18.6)	18.6 (15.7-26.9)
Serum Creatinine（µmol/L)	133.6 (92.7-195.7)	132.9 (92.5-248.1)	160.1 (82.0-227.0)	307.1 (224.4-409.3)

Values represent medians with the corresponding interquartile range

### ELISA for sCD100 analysis

Our group has previously established seven hybridoma cell lines secreting monoclonal antibodies to human CD100 [20] by immunizing mice with eukaryoticly expressed CD100 protein and routine B lymphocyte hybridoma technique. Using these mAbs, an optimized sandwich enzyme-linked immunosorbent assay (ELISA) was established to detect sCD100 in biological fluids including human plasma, serum and cell culture supernatant. Briefly, ELISA plates (96 wells, Corning Inc, USA) were coated with FMU-CD100-2.4 mAb (10µg/ml in 0.05M Na _2_CO_3_-NaHCO_3_, pH 9.6) overnight at 4°C. After washing, the wells were blocked with assay buffer (5% newborn calf serum in PBS) for 1h at room temperature. Human CD100-his (CD100-his) (Vaccinex Inc, USA) was diluted with assay buffer to set 7 points of diluted standard (50, 25, 12.5, 6.25, 3.125, 1.5625 and 0.78125 ng/ml), and a blank was also included. The standard solution or plasma samples were added to the wells (100µl/well) at 37°C for 1h. After washing, HRP (horseradish peroxidase)-conjugated FMU-CD100-2.3 mAb in assay buffer as a detecting antibody was added and incubated at room temperature for 1 h. TMB (3, 3', 5, 5'-Tetramethylbenzidine)-substrate (eBioscience Inc, USA) was added to the wells and incubated for 30 min in dark. Absorbance was measured at 450 nm by a plate-reader (Bio-Rad, Hercules, CA, USA).

### Immunoprecipitation and Western Blotting

CD100 mAb FMU-CD100-2.3 (10 mg) was coupled to 0.5g of CNBr-activated Sepharose 4B beads (#17-0430-01, GE healthcare, USA) according to the manufacturer’s protocol. 200µl of each plasma sample diluted in 1.2ml PBS was incubated with 50µl of CD100 mAb coulped beads at room temperature for 2h. After washing with PBS, the immunoprecipitate of the sCD100 and CD100 mAb was eluted from the beads by boiling for 3 min in loading buffer (100 mM Tris-HCl pH 6.8, 4% SDS, 20% glycerol, 0.2% bromophenol blue, and 5% 2-mercaptoethanol). The supernatant proteins were separated by SDS-PAGE and electrotransferred on nitrocellulose membrane. The membrane was blocked with blocking buffer (Tris-buffered saline and 0.1% Tween 20 with 5% nonfat dry milk) for 1 h and then probed with CD100 rabbit mAb (#3134-1, Epitomics Inc, USA) overnight at 4°C. The membrane was developed using HRP-conjugated secondary antibodies and enhanced chemiluminescence.

### Flow Cytometry

CD100-FMU-2.3 mAb was labeled with APC using DyLight™ Antibody Labeling Kits (#84535, Thermo Inc, USA). FITC-CD19, PE-CD4, PE-CD8 and PE-CD14 were purchased from BD (San Diego, USA). PerCP-cy5.5-CD3 and isotype control antibodies were purchased from Biolegend (San Diego, USA). PBMCs were isolated from anti-coagulated blood by Ficoll gradient centrifugation. The freshly isolated PBMCs of the patients (approximately 2×10^6^ cells/ml) were incubated with antibodies for 30 min at 4°C in the dark. The multiple-color combinations were following: CD4-PE/CD3-PerCP-Cy5.5/CD100-APC, CD19-FITC/CD14-PE/CD100-APC, and CD8-PE/CD100-APC. FITC, PE, PerCP-Cy5.5 and APC labeled normal mouse IgG was used as isotype control. After an additional wash with wash buffer (5% FCS and 0.2% NaN_3_ in PBS), the cells were resuspended in fixing buffer (2% glucose, 1% paraformaldehyde and 0.02% NaN_3_ in PBS) before the FCM analysis was performed. A minimum of 20,000 cells were acquired for analysis and the cells were acquired on BD FACS Calibur. The data were analyzed using the FlowJo 5.7.1 software.

### Statistical Analysis

The analysis was performed by SPSS (version13) and GraphPad Prism5 software. Continuous variables were presented as medians with corresponding interquartile ranges (IQR). The significance of the differences between different groups was determined by the Mann–Whitney U test. The multivariate models (Spearman correlations and multiple linear regression analysis) were performed to identify the association between clinical parameters and elevated sCD100 levels. Soluble CD100 levels were used as a dependent variable (Y) and the clinical parameters (X1) and age (X^2^) were used as independent variables. After the forcing of age into the model, the following independent variables were considered for the model: platelet count (PLT), white blood cell count (WBC), serum creatinine level (Crea) and blood urea nitrogen level (BUN). Only variables that had a P≤0.05 value were considered in the final fitted model. A value of P<0.05 (2-sided) was considered statistically significant.

## Results

A total of 99 HFRS patients were confirmed to have HTNV infection by detection of IgM or IgG specific antibodies to HTNV in the patients’ serum specimens. Overall, 195 plasma samples were collected at the febrile/hypotensive (Febr/Hypo), oliguric (Olig), diuretic (Diur), and convalescent (Conv) stages of the disease. According to the clinical records and diagnostic criteria, 17, 25, 29, and 28 patients were diagnosed respectively as having mild, moderate, severe, and critical HFRS. The details of the clinical parameters detected during the hospitalization of the patients were summed in Table 1.

**Figure 1:**
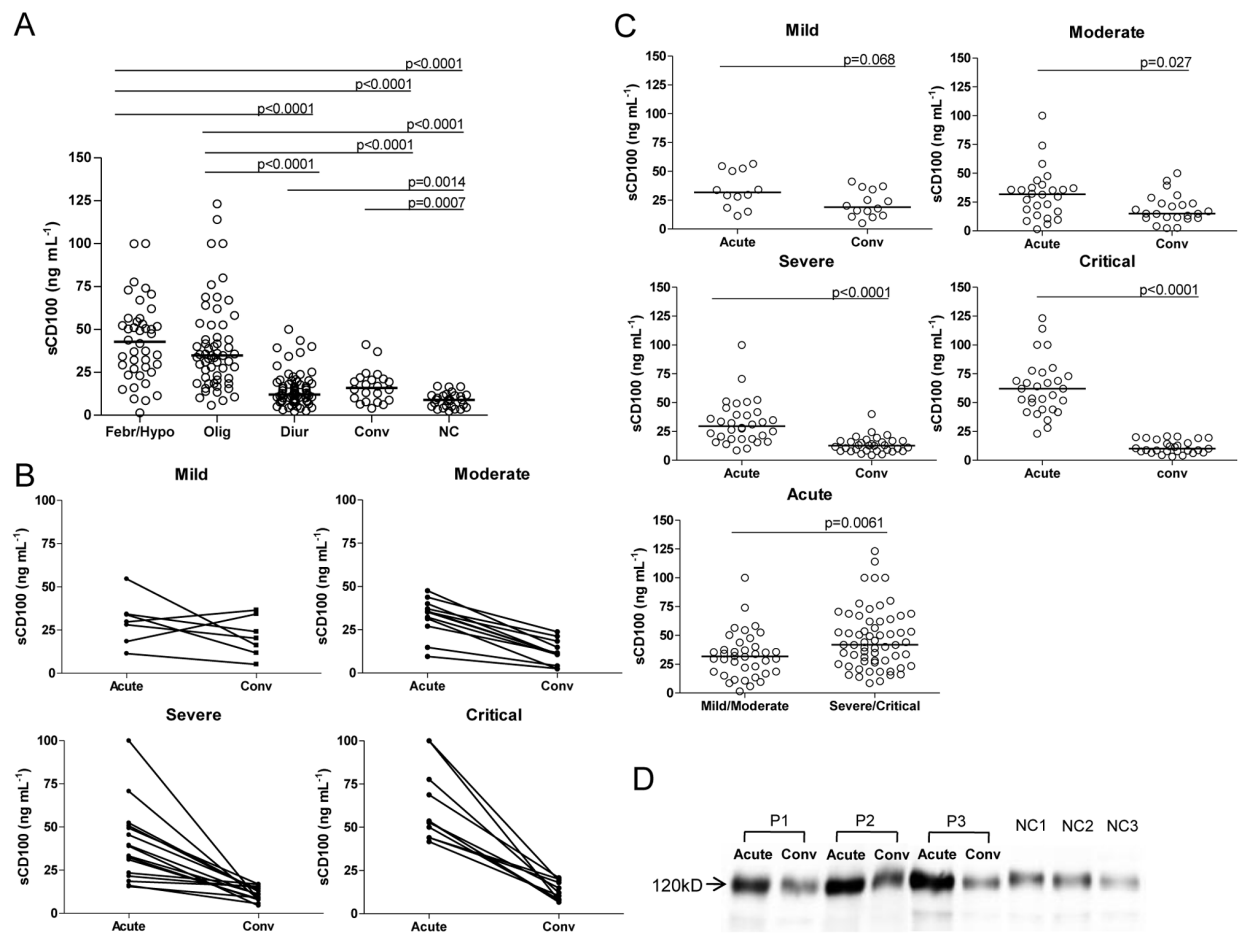
The kinetic changes of plasma soluble Sema4D/CD100 (sCD100) levels in different severity groups of HFRS patients. (A) Comparison of plasma sCD100 levels in different stages of HFRS patients. Data were obtained from 195 plasma samples collected at the febrile/hypotensive (Febr/Hypo), oliguric (Olig), diuretic (Diur), and convalescent (Conv) stages of HFRS patients and from 27 plasma samples of healthy subjects as normal control (NC). Significant differences were found for febrile/ hypotensive or oliguric vs. NC (P< 0.0001); febrile/ hypotensive vs. diuretic or convalescent (P< 0.0001); oliguric vs. diuretic or convalescent (P< 0.0001); diuretic vs. NC (P= 0.0014), convalescent vs. NC (P= 0.0007). (B) The changes of plasma sCD100 levels in acute phase (including febrile, hypotensive, or oliguric stage) and convalescent phase (including diuretic or convalescent stage) of the same individual in different severity groups of the disease. (C) The changes of plasma sCD100 levels in acute phase and convalescent phase in different severity groups of the disease and comparison of acute phase sCD100 level of different disease sverities. (D) Comparison of sCD100 levels in acute phase (including febrile, hypotensive, or oliguric stage) and convalescent phase (including diuretic or convalescent stage) of HFRS and normal control analyzed by Western Blot (representative results of 3 HFRS patients and 3 healthy subjects). The significance of the differences between different groups was determined by the Mann–Whitney U test. Black lines represent medians and P values are plotted in each graph.

The median sCD100 levels for febrile/ hypotensive, oliguric, diuretic and convalescent stages were 42.8 ng/ml, 34.8 ng/ml, 12.1 ng/ml and 15.9 ng/ml, respectively. According to clinical classification criteria for the disease course, the acute phase includes febrile, hypotensive and oliguric stages, and the convalescent phase includes diuretic and convalescent stages. In acute phase, the plasma sCD100 level in HFRS patients was significantly higher than that in normal controls (febrile/ hypotensive or oliguric vs. NC, P < 0.0001). The plasma sCD100 level of HFRS patients decreased in convalescent phase (febrile/hypotensive vs. diuretic or convalescent; oliguric vs. diuretic or convalescent, P< 0.0001) but still higher than of normal controls (diuretic vs. NC, P= 0.0014), (convalescent vs. NC, P = 0.0007) (Figure 1A). Patients with different disease severity showed the same tendency of plasma sCD100 changes, but more dramatic decline in patients in severe/critical group (Figure 1B and 1C, *P*<0.0001). When plasma sCD100 concentrations in mild/moderate group were compared with those in severe/critical group, only 7 (18.4%) of the 38 cases in mild/moderate group had plasma sCD100 levels over 50 ng/ml, while 26 (42.6%) of the 61 cases in severe/critical group had sCD100 levels over 50 ng/ml (2.32 fold high vs. mild/moderate group). These results indicated that there would be some kind of association between the plasma sCD100 level and the disease severity during the course of HFRS. Western blot assay of plasma sCD100 also showed that the sCD100 level was markedly higher in acute phase than in convalescent phase. The molecular weight of the sCD100 in plasma from both patients and normal controls is 120 kDa in the reduced condition which is consistent with the size of extracellular region of CD100 (Figure 1D).

**Figure 2:**
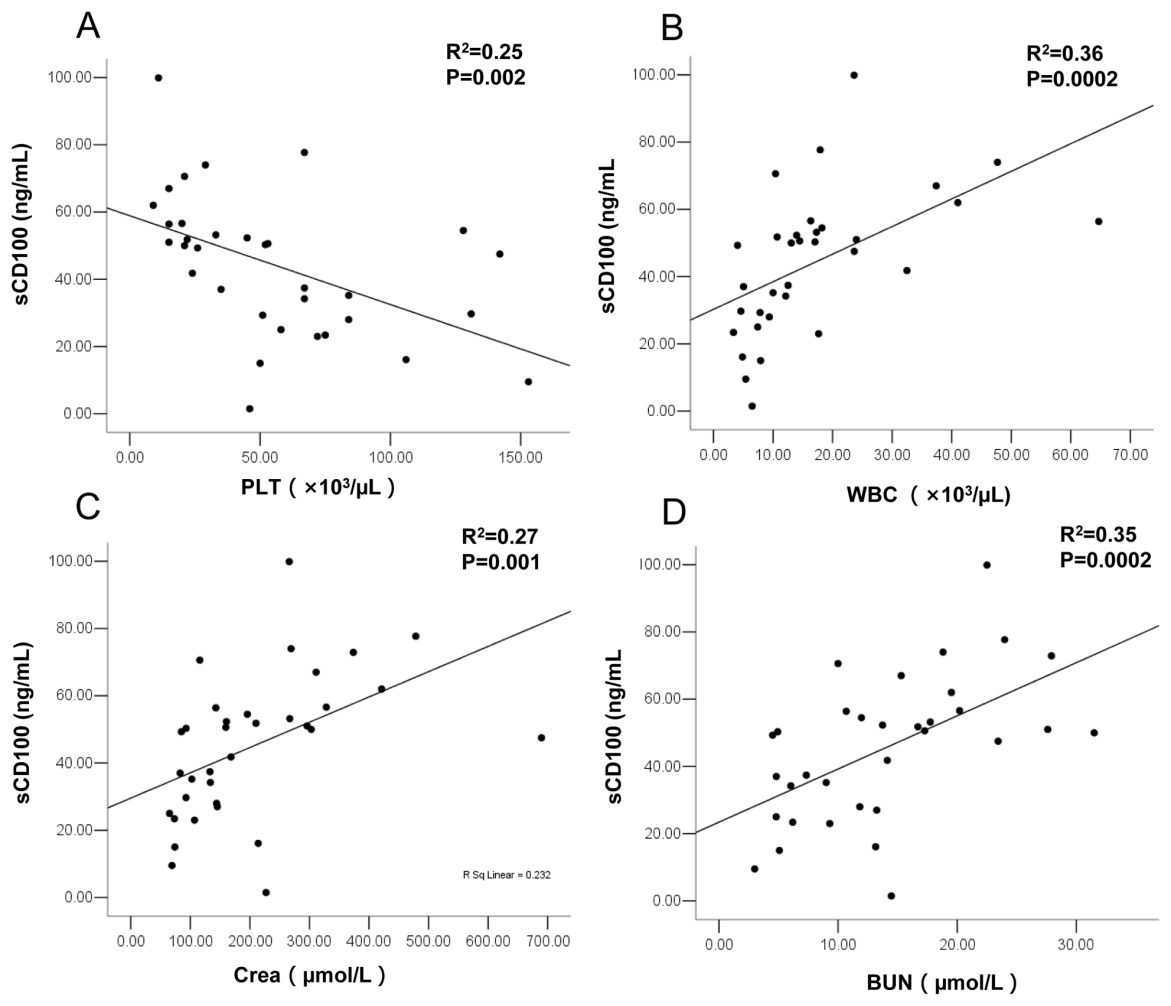
Relationship between elevated plasma sCD100 level and clinical parameters. The values derived from the same blood sample in febrile or hypotensive stage at the time of admission. Multivariate linear regression analysis of plasma sCD100 level (dependent variable) with clinical parameters (X1) and age (X^2^) as independent variables were performed. Platelet count (PLT) (A), white blood cell count (WBC) (B), serum creatinine level (Crea) (C), and blood urea nitrogen level (BUN) (D) (n=33) were shown. The adjusted R^2^ and P value are plotted in each graph.

The relationships between plasma sCD100 levels at febrile or hypotensive stages (the time of admission generally about 3-7 days after the onset of disease) and the four clinical parameters that could represent the severity of the disease were analyzed. The results showed that age was not correlated with sCD100 levels, PLT was inversely associated with sCD100 levels and WBC, Crea and BUN were positively associated with sCD100 levels. Multivariate linear regression analysis showed that by adjusting for age, PLT (adjusted R^2^ =0.25, P=0.002), WBC (adjusted R^2^ =0.36, P=0.0002), Crea (adjusted R^2^ =0.27, P=0.001) and BUN (adjusted R^2^ =0.35, P=0.0002) contributed independently to the elevated sCD100 levels (Figure 2).

**Figure 3:**
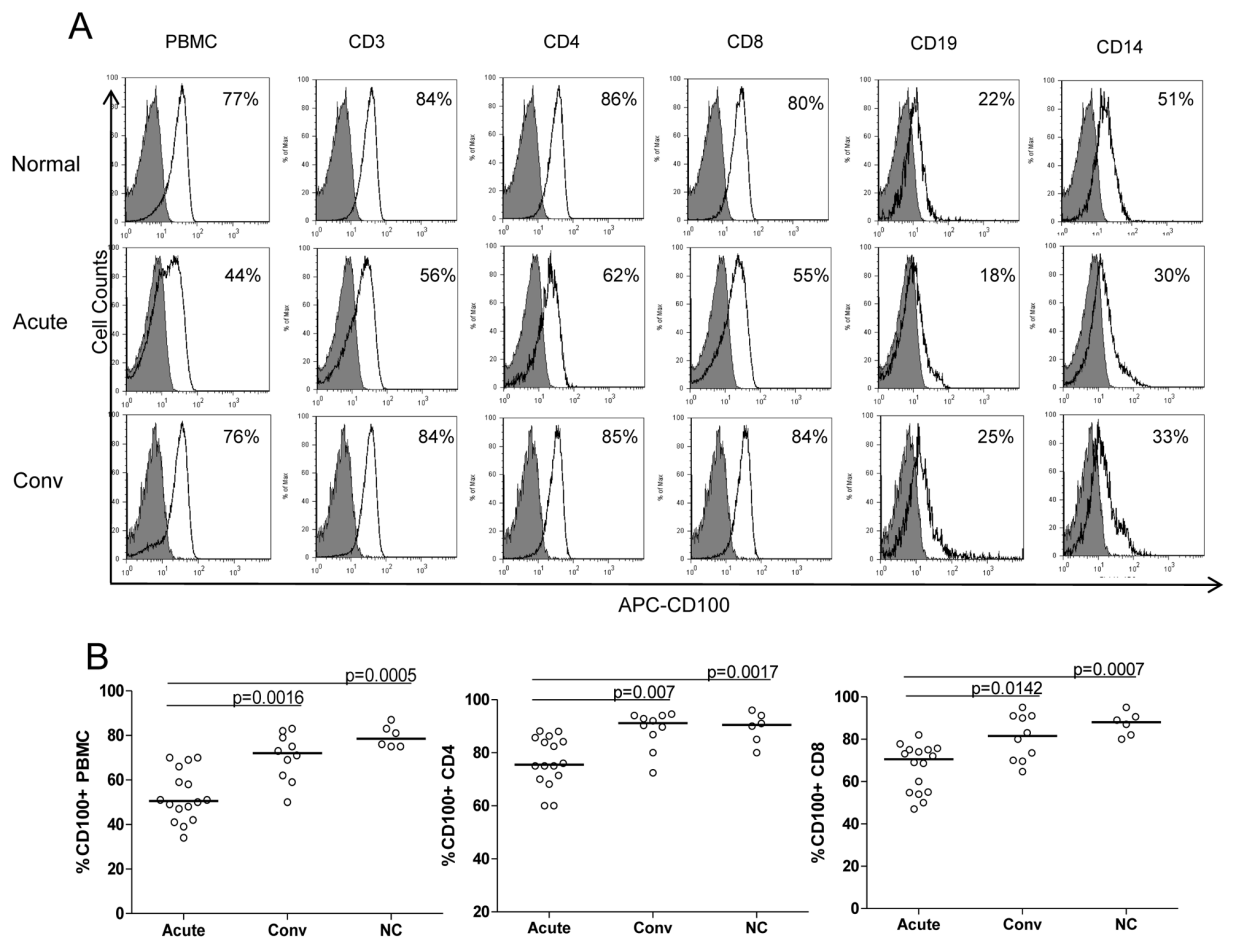
Membrane CD100 expression on PBMC of HFRS patients compared with that of normal subjects. PBMC samples were collected in the acute phase (including febrile, hypotensive, or oliguric stage) and convalescent phase (including diuretic or convalescent stage) of HFRS patients and 6 normal controls. PBMCs were stained using the following multiple-color combinations: CD4-PE/CD3-PerCP-Cy5.5/CD100-APC, CD19-FITC/CD14-PE/CD100-APC, CD8-PE/CD100-APC. FITC, PE, PerCP-Cy5.5, and APC labeled normal mouse IgG was used as isotype control. (A) Representative results of membrane CD100 expression on different subpopulations of PBMC in acute phase and convalescent phase. Shaded histograms represent isotype control and black lines represent membrane CD100 expression. (B) Comparison of membrane CD100 expression on PBMC, CD4^+^ T cells and CD8^+^ T cells. The significance of the differences between different groups was determined by the Mann–Whitney U test. Black lines represent medians and P values are plotted in each graph.

The membrane CD100 on PBMCs was also investigated. The results showed that membrane CD100 levels were more highly expressed on T cells than the levels on B cells and monocytes. The expressions of membrane CD100 on PBMCs including CD4^+^ T cells, CD8^+^ T cells, B cells and monocytes in HFRS all decreased to different extents in the acute phase compared with those in normal controls, and the expressions almost recovered in the convalescent phase (Figure 3A). On the major subpopulations of PBMCs, CD4^+^ T cells and CD8^+^ T cells, the decrease of membrane CD100 expression presented statistically difference (Figure 3B).

## Discussion

Our study demonstrated that the plasma sCD100 level in acute phase of the HFRS patients was significantly higher than its corresponding level in healthy controls. Importantly, higher plasma sCD100 levels in acute phase were associated with a higher severity of the disease. The elevation of plasma sCD100 levels was also correlated significantly with the disease severity-indicating clinical parameters.

Although the generation of sCD100 is still not clear, it is generally considered that sCD100 could be released from the immune cells. In this study, we reported the molecular weight of sCD100 from the fluid of the human body at disease condition is consistent with the size of the extracellular region of CD100, indicating that plasma sCD100 could be a proteolytically-cleaved product. Some previous studies showed that the shedding of sCD100 is strictly dependent on a proteolytic cascade after cellular activation [10,21] and T cells are the major CD100-producing cells in the immune system. CD100 expressed on T cells could interact with DCs to promote their activation and maturation which in turn enhanced T-cell activation [22]. CD100 was also reported to be associated with protein tyrosine phosphatase CD45. This association was enhanced during T-cell activation and down-regulated CD100 molecules at the cell surface [23]. It is possible that the immune cellular activation and the increased activity of a metalloprotease caused by HTNV infection may promote the shedding of sCD100 from PBMCs (especially from T cells) in HFRS patients, thus contributing to the elevated levels of plasma sCD100. Therefore, the high level of sCD100 might reflect the activated status of adaptive immune response and might act as an indicator for the development of HFRS. As cellular activation is a general aspect of immune response and cellular activation can regulate both the expression of mCD100 and the release of sCD100, it is highly possible that the elevated sCD100 may also be an indicator for other infectious diseases. We assumed that any disease with an immunopathological manifestation might display the abnormality of CD100 level. Further studies on the functional role of CD100 expression in other acute infectious diseases are therefore suggested.

Some biological functions have been reported to be mediated by sCD100. The increased levels of CD100 were detected in cerebrospinal fluid and spinal cords of patients with HTLV-1 associated myelopathy [16] and in animal model of mice immunized with a T cell dependent antigen and MRL/lpr mice [10]. However, it was not clear whether the elevated sCD100 was involved in the progression of these diseases. The positive correlation found in this study between the elevation of plasma sCD100 levels and the disease severity-indicating clinical parameters suggests that sCD100 might be involved in the pathogenesis of HFRS for the following two reasons. First, CD100 might participate in immune renal injury in a tissue-specific manner. CD100 has been shown to have a pathogenic role in a model of experimental glomerulonephritis induced by circulating immune complexes. In CD100^-/-^ mice, the glomeruli exhibited milder histological changes compared with (the more severe changes in) WT mice. The antigen speciﬁc immune responses were also reduced in CD100^-/-^ mice, including IFN-γ and IL-4 production, T and B cells activation, macrophage and CD4^+^ cell inﬁltration, IgG, and C3 deposition in Glomerular [24,25]. These results have implied that CD100 enhances nephritogenic immune responses. In case of HFRS, renal injury was proven to be driven by the deposition of immune complexes in glomeruli [4,26]. It is known that both CD100 and the tissue receptor for CD100, plexin-B1, are expressed in the kidney [24]. It is probably that after shedding from activated immune cells caused by HTNV infection, sCD100 may remain in circulation to promote B cell proliferation and immunoglobulin production, and enhance inflammatory response caused by T cell-APC interaction, all of which, to some extent, may lead to nephritogenic immune injury. Second, sCD100 could regulate the integrity of endothelium and microvascular permeability. Endothelial cells form the primary fluid barrier of the vasculature and are the determinants of capillary integrity and permeability. Although HTNV infection does not lyse infected endothelial cells, it might play the primary role on endothelial cells for altering vascular permeability leading to the pathogenesis [26]. Soluble CD100 is highly angiogenic through its high affinity receptor Plexin-B1 on endothelial cells, leading to cytoskeletal reorganization [27–30]. The cytoskeletal remodeling process serves to regulate endothelial cell adherence junction assembly and disassembly process, resulting in the enhanced endothelium permeability. Furthermore, CD100 shares certain signaling pathway downstream with VEGF and cooperates with VEGF to promote angiogenesis [31]. Our previous research has found high levels of plasma VEGF in HFRS patients [32]. Thus, it seems that the pro-permeability effects are amplificated under the synergistic effect of sCD100 and VEGF in contributing to plasma leak and microvascular hemorrhage syndrome in HFRS patients. Though we cannot rule out that the enhanced sCD100 is a consequence, rather than a cause, of the more severe condition of the disease, it is reasonable that the high level of sCD100 may reflect the highly activated status of the adaptive immune system. Apparently, further studies are necessary for a better understanding of the underlying mechanisms of CD100 involving in the HFRS, which may be helpful for us to further clarify the pathogenesis of the disease and to provide some useful information for HFRS prevention and treatment.

Overall, we reported for the first time the level of plasma sCD100 in HFRS patients. The elevated sCD100 in plasma seems to be an important indicator for the development of HFRS. Our results provided some evidence on a possible association between the increased release of sCD100 and different disease severities in HFRS. Further studies need to be conducted for the roles of CD100 in both humoral immune response and cellular immune response after HTNV infection and for a better understanding of the function of CD100 in the pathogenesis of HFRS.
